# Modulation of TRPV1 on Odontoblast-like Cells Using Capsazepine-Loaded Nanogels

**DOI:** 10.3390/pharmaceutics16030355

**Published:** 2024-03-03

**Authors:** Lilia Jadith Bernal-Cepeda, Ronald Andrés Jiménez, Myriam L. Velandia-Romero, Paola Acosta-Guzmán, Jaime E. Castellanos

**Affiliations:** 1 IBAPO Group, School of Dentistry, Universidad Nacional de Colombia, Bogota 111321, Colombia; ljbernalc@unal.edu.co; 2 INQA Group, Pharmaceutical Chemistry Program, Department of Chemistry, Universidad El Bosque, Bogota 110121, Colombia; rajimenez@unbosque.edu.co (R.A.J.); pacostagu@unbosque.edu.co (P.A.-G.); 3 Virology Group, Vice-Chancellor of Research, Universidad El Bosque, Bogota 110121, Colombia; velandiamyriam@unbosque.edu.co; 4 Department of Chemistry, Universidad Nacional de Colombia, Bogota 111321, Colombia

**Keywords:** nanogel, dental pain, transient receptor potential channel vanilloid 1, capsazepine

## Abstract

The modulation of TRPV1 emerges as a promising strategy for dental pain management. This study aimed to assess TRPV1 modulation in a human odontoblast-like cell model using Capsazepine (CZP) loaded in a nanogel delivery system. Gelatin nanogels, synthesized via the emulsification-gelation technique, were characterized and loaded with the TRPV1 antagonist, CZP. HPLC determined a remarkable 67.5 ± 0.04% CZP loading efficiency, with 71.7% of nanogels falling within the 300–950 nm size range, as evidenced by light microscopy. Moreover, CZP-loaded nanogels had a low cytotoxicity. An FTIR analysis showed no adverse chemical interactions, ensuring stability and active release. When examining biological responses, TRPV1 expression and channel activity were assessed in odontoblast-like cells. On the fifth day post-treatment, cells treated with CZP-loaded nanogels exhibited an increased TRPV1 expression and a reduction in calcium fluxes after agonist stimulus (F/F0 ratio 1.18 ± 0.18), resembling the response in free CZP-treated cells (1.28 ± 0.15). A two-way analysis of variance and the Tukey’s test were used to determine statistical significance (*p* < 0.05). This delivery system, proven to be economical and straightforward, holds promise for dental pain management and potential local use. Local administration minimizes systemic adverse effects, making it a practical solution for releasing molecules in the oral cavity.

## 1. Introduction

Dental pain is the leading cause for patients to consult dental professionals [1]. Although some therapeutic alternatives exist, the analgesic effect is not always adequate, highlighting the need to explore novel pharmacological strategies and local application formulations to effectively alleviate pain. One such strategy is the modulation of transient receptor potential (TRP) channels, which have been studied for their potential role in pain management [2] and association with other alterations in the oral cavity [3]. Among these channels, transient receptor potential vanilloid 1 (TRPV1) has been extensively investigated for its participation in various physiological and pathological processes, and several studies have focused on developing small-molecule modulators targeting this channel to manage pain, including nociceptive and neuropathic pain [4].

Several TRPV1 agonists and antagonists have been employed for pain management and some molecules are currently in clinical phases, utilizing both local and systemic administration routes [5]. One such example is capsazepine (CZP; N-[2-(4-chlorophenyl)ethyl]-1,3,4,5-tetrahydro-7,8-dihydroxy-2H-2-benzazepine-2 carbothioamide), a synthetic analog of capsaicin that functions as a competitive antagonist of TRPV1. CZP has been used in in vivo studies for the management of different types of pain [6,7], and its therapeutic use has been proposed for the treatment of major disorders such as cancer, colitis, pancreatitis, malaria, and epilepsy [8]. Additionally, the synergistic effect of CZP and opioid analgesics has been reported to improve the antinociceptive effect and prevent the development of opioid tolerance and physical dependence [9]. This underscores the importance of TRPV1 modulation as a significant therapeutic strategy.

Another challenge in dental pain management is the lack of pharmaceutical formulations that are tailored for local use in the oral cavity. This challenge arises due to issues such as low concentrations and poor adherence at the site of action, the presence of fluids, (e.g., saliva and crevicular fluid), and adverse reactions at the administration site [10,11]. Nevertheless, controlled release systems, such as nanocarriers, have been suggested as a strategy to address these challenges in the management of various pathologies, including pain and tooth sensitivity. These systems offer advantages such as a reduced frequency of administration, increased adherence to treatment, prolonged and constant therapeutic effects, reduced adverse effects, drug protection against environmental conditions, increased bioavailability, and biological half-life [12]. These advantages can be harnessed in the oral cavity to effectively manage various common signs and symptoms in the population.

In recent years, nanocarriers of organic and inorganic origins have gained significant attention as controlled release systems for drugs [13]. Some examples include metallic and polymeric nanoparticles, micelles, fullerenes, graphene, liposomes, dendrimers, quantum dots, gold, mesoporous silica nanoparticles, and nanogels. Nanogels, defined as hydrogel nanoparticles with high water content and sizes ranging from 1 to 1000 nm, are formed by physically or chemically cross-linked networks [14]. These systems allow for the encapsulation of small molecules (e.g., drugs) or macromolecules, including proteins and nucleic acids. Due to their physicochemical characteristics, nanogels are promising therapeutic delivery systems [15]. Although nanogels can be obtained using synthetic polymers, they can also be synthesized from natural biopolymers like gelatin, which possesses characteristics of great interest, such as a low cost, easy availability, biodegradability, low antigenicity, and biocompatibility, and has been used for the development of nano- and microgels with potential use in medical applications [16,17], including dental pain management.

There are a few reports on nanogels for local use in the oral cavity, including their use for remineralization in dental caries processes [18,19] and the management of periodontal disease [20]; furthermore, there are many aspects that remain unknown. Therefore, this study aimed to develop and characterize a novel gelatin-based nanogel delivery system loaded with CZP that has not been reported previously, and evaluate its ability to modulate TRPV1 channels in an in vitro oral cell model as a potential therapeutic strategy for dental pain management. In the context of this study, the null hypothesis asserts that there is no statistically significant distinction in the modulation of TRPV1 channels observed in human odontoblast-like cells (OLC) when comparing the utilization of CZP-loaded nanogels and the conventional administration of free CZP.

## 2. Materials and Methods

### 2.1. Prototype Nanogel Formulation

The formulation of different nanogel prototypes using gelatin type B (MW = 40,000–50,000 g/mol, Merck Millipore, Burlington, MA, USA) was achieved using an emulsification-gelation technique [21]. A water-in-oil emulsion was prepared using 0.25 g of gelatin in the dispersed phase and a variable volume of purified water [22]. This phase was heated at 37 °C for 5 min under constant magnetic stirring until a colloidal dispersion was obtained, and then adjusted with 0.1% glutaraldehyde (Merck Millipore, Burlington, MA, USA) as a chemical crosslinker. The continuous phase consisted of 25 g of white mineral oil USP (Ciacomeq, Bogota, Colombia), and the concentration of sorbitan trioleate (Merck Millipore, Burlington, MA, USA) as a surfactant was adjusted for different prototypes (Table 1). This phase was immersed in an ice bath at 0 °C and subjected to constant magnetic stirring at 1000 rpm. The dispersed phase was then slowly added dropwise into the continuous phase, and the droplets were homogenized under various ultrasonic homogenization conditions (Qsonica Sonicator Q500, Newtown, CT, USA). Subsequently, some prototypes were homogenized at 13,800 rpm for 15 min using a rotor-stator homogenizer (MiniBatch D-1, MICCRA, Buggingen, Germany). Finally, the prototypes were stored at −20 °C for 16 h to induce the sol–gel transition. The specific conditions of the different samples are presented in Table 1.

The suspension was then purified by heating to room temperature. The mixture was centrifuged at 2000× *g* for 10 min using a Rotina 380R refrigerated centrifuge (Hettich, Tuttlingen, Germany). Mineral oil residues were removed and three washes were performed with a 1:1 ethanol-H_2_O solution for 15 min, followed by centrifugation at 2000× *g* for 10 min and supernatant removal. Finally, three washes were conducted with sterile-filtered 1× PBS and antibiotics/antimycotics (penicillin 100 U/mL, streptomycin 100 μg/mL, and amphotericin 0.25 μg/mL; Biowest, Nuaillé, France) for 15 min, and the nanogels were processed under sterile conditions. They were resuspended in 500 μL of sterile-filtered PBS, treated with ultraviolet radiation of 254 nm for 15 min in a PCR cabinet (ESCO, Singapore), and stored at 4 °C until loading and characterization. The nanogels were fabricated in triplicate and the yield (%) was calculated using the equation: (amount obtained experimentally/theoretical amount) × 100.

To evaluate the sterility of the nanogels, a drop of the CZP-loaded and unloaded nanogels was spread over the surface of an agar plate and incubated at 37 °C for 48 h. The bacterial culture media used were brain heart infusion nutrient agar and MacConkey agar in Petri dishes.

### 2.2. Preparation of CZP-Loaded Nanogels and Loading Efficiency

CZP (Tocris, Bristol, UK) reconstituted in dimethyl sulfoxide (DMSO; Merck Millipore, Burlington, MA, USA) was used as the active compound, and working solutions were developed using sterile water. CZP loading was carried out using sorption (adsorption and desorption) [23], and the loading concentration was determined using a previously established 50% cytotoxic concentration of CZP on OLCs [24]. Taking into account the burst effect of these delivery systems, which corresponds to 20–30% of the initial load, the nanogels were loaded with 133 μM of CZP in sterile water. The loading process involved mixing 100 mg of nanogels with 1.5 mL of CZP in 2 mL tubes, followed by constant horizontal stirring at 100 rpm and 20 °C for 24 h using an orbital Shaker OS-20 (BOECO, Hamburg, Germany). After loading, the nanogels were centrifuged at 1500× *g* using a refrigerated microcentrifuge (Eppendorf, Hamburg, Germany) and the supernatant was removed and stored at −20 °C for determination of the loading efficiency. The loading efficiency was determined using HPLC and the equation:

Loading efficiency (%) = [Concentration of CZP loaded in the nanogels/concentration of CZP in the loading solution] × 100.

An in vitro enzymatic degradation assay was also performed. Briefly, 100 mg of CZP-loaded nanogels was treated with 0.5% trypsin in sterile PBS for 6 h at 20 °C to induce gelatin degradation and CZP release. The tube was then centrifuged at 1500× *g* for 10 min and the supernatant was used for CZP quantification using HPLC. The experiments were performed in triplicate.

### 2.3. High-Performance Liquid Chromatography

The protocol described by Gao et al. [25] was used with some modifications. Briefly, the separation of CZP from the samples was performed on a Shimadzu HPLC instrument (Prominence-i model LC 2030, Kyoto, Japan) using an octadecylsilane column (4.6 × 50 mm, 3 µm particle size; Shimadzu, Kyoto, Japan). The isocratic mobile phase consisted of 60:40 water/acetonitrile (Merck Millipore, Burlington, MA, USA). The flow rate was 1.0 mL/min, the injection volume was 10 μL at 25 °C, and the elution of CZP from the HPLC column was monitored by UV absorption at 234 nm for 10 min. Different dilutions of CZP in artificial saliva or water (20, 50, 80, 110, 140, and 170 μM) were used to develop a concentration curve using the measured absorbance and areas. The curve allowed for CZP quantification in different samples, and the loading efficiency and release profile were calculated. Chromatograms were analyzed using the Shimadzu LabSolutions software, version DB V.6.106. The experiments were performed in triplicate.

### 2.4. Nanogel Size Measurement

The sizes of the CZP-loaded and unloaded nanogels were characterized and evaluated using light microscopy. A drop of the nanogel, resuspended in sterile PBS, was placed on a slide and visualized using a microscope ZEISS Axio Imager M2 with the Zen 12 software and a 63× objective (Oberkochen, Germany). Image analysis was performed using the ImageJ2 software, version 2.14.0. The size and distribution of the nanogels are expressed as mean ± standard deviation, and the polydispersity index (PDI) was determined using the formula (σ/d)^2^, where PDI = the square of the standard deviation divided by the mean particle diameter [26]. A total of 120 particles were analyzed per field.

### 2.5. Fourier Transform Infrared Spectroscopy (FTIR) and Zeta Potential

The CZP-loaded and unloaded nanogels were dried at 37 °C for 7 days, and the functional groups of the CZP powder and the nanogels were determined by FTIR using attenuated total reflectance on an ALPHA II Compact FT-IR Spectrometer (Bruker, Billerica, MA, USA). Thirty scans were averaged per spectrum, and the images were obtained covering the wavenumbers 4000–400 cm^−1^ with a nominal spectral resolution of 8 cm^−1^. Data acquisition and spectral analyses were performed using the spectroscopy software OPUS, version 6.5 (Bruker, Billerica, MA, USA). In addition, the Z potential of the CZP-loaded and unloaded prototypes dispersed in distilled water was determined using Litesizer 500 equipment (Anton Paar, Graz, Austria). The samples were placed in clear cells (Omega Cuvette; Anton Paar, Graz, Austria) and the results were recorded and analyzed using the Kalliope software, version 3.2.5 (Anton Paar, Graz, Austria). The assays were performed in triplicate.

### 2.6. In Vitro Release of CZP-Loaded Nanogels

The in vitro release of CZP was determined using artificial saliva as the release medium. For its preparation, 0.103 g/L of CaCl_2_, 0.019 g/L of MgCl_2_∙6H_2_O, 0.544 g/L of KH_2_PO_4_, and 2.24 g/L of KCl were used, and buffer (TCP-KOH) was added to adjust the pH to 7.0 [27]. Briefly, 100 mg of CZP-loaded nanogels was placed in 1000 μL of artificial saliva and subjected to constant horizontal agitation at 100 rpm and 20 °C using a horizontal orbital. At 0, 6, and 12 h, and daily for 8 days, 500 μL of the medium was withdrawn and stored at −20 °C until processing using HPLC. Each time, the withdrawn volume was replaced with 500 μL of new artificial saliva. As a control, unloaded nanogels were subjected to identical treatment.

### 2.7. Cell Culture

OLCs and primary human gingival fibroblasts (HGFs) were cultured using Dulbecco’s modified Eagle’s medium (DMEM; Biowest, Nuaillé, France), supplemented with 10% fetal bovine serum (FBS; Biowest, Nuaillé, France), 100 U/mL of penicillin, 100 μg/mL of streptomycin, and 0.25 μg/mL of amphotericin. In the OLC culture, the medium also contained 10 ng/mL of TGF-β1 (Abcam, Cambridge, UK). These cells were previously isolated from consenting healthy donors. In the case of OLCs, their characterization included assessing dentin sialophosphoprotein and dentin matrix acidic phosphoprotein expression [24], while for HGF, vimentin expression was evaluated.

### 2.8. Cytotoxicity Evaluation

Cell viability was assessed using the resazurin assay (Merck Millipore, Burlington, MA, USA). OLCs and HGFs were seeded in 96-well plates (SPL Life Sciences, Pocheon, Republic of Korea) at a density of 2 × 10^4^ cells/well using supplemented DMEM.

After 24 h of adherence at 37 °C and 5% CO_2_, the cells were treated with elutions of 0.25, 0.5, 1, and 2 mg/mL of CZP-loaded and unloaded nanogels diluted in DMEM supplemented with the respective components. Subsequently, the treated cells were processed daily for seven days. The leachate was removed and the cells were incubated with 4.4 μM of resazurin in DMEM with no FBS for 3 h at 37 °C and 5% CO_2_. Fluorescence was measured at wavelengths of 530 nm/590 nm using a microplate reader (CLARIOstar Plus BMG Labtech, Ortenberg, Germany), and cytotoxicity was calculated using GraphPad Prism 8 (La Jolla, CA, USA). For cytotoxicity controls, cells treated with DMEM + 2% DMSO and untreated cells were used as positive and negative controls, respectively. The cell viability (%) was calculated using the formula: [relative fluorescence unit (RFU) of treated cells/RFU of untreated cells] × 100.

### 2.9. Biological Activity Assays of Nanogels in OLCs

To assess the response induced by free CZP or its active release from nanogels in the OLCs, RT-qPCR, quantification, and immunofluorescence for TRPV1 were conducted at various time points post-stimulation. TRPV1 channel activity was determined by measuring the calcium influx using a Fluo-4 AM probe (Thermo Fisher Scientific, Waltham, MA, USA) after capsaicin stimulation (CAP; Merck Millipore, Burlington, MA, USA). As a control, OLCs treated with 40 μM of free CZP were used and untreated cells served as the negative control. OLCs were seeded in 35 mm dishes at a density of 5 × 10^4^ cells/dish and treated with CZP-loaded and unloaded nanogels at 37 °C and 5% CO_2_. Total RNA was extracted from the monolayer at different time intervals following treatment with TRIzol reagent (Invitrogen, Waltham, MA, USA) and quantified by spectrophotometry. The RNA was then used to evaluate the presence of *TRPV1* transcripts using SYBR Green (Luna^®^ Universal qPCR Master Mix; New England Biolabs, Ipswich, MA, USA) and specific primers [28]. Relative quantification was performed using Schefe’s formula [29], taking into account the amplification cycles and efficiencies obtained using the LinRegPCR software, version 2021.2, with specific primers for *CRN* and *GAPDH* as reference genes (Table 2) [30].

Simultaneously, OLCs were seeded on 10 μg/mL poly-L-lysine-processed coverslips at a density of 2 × 10^3^ cells/coverslip. Cells treated on different days were fixed with 4% paraformaldehyde (PFA; Carlo Erba, Emmendingen, Germany) and used to perform indirect immunofluorescence with an anti-TRPV1 polyclonal antibody (Abcam, Cambridge, UK) at a dilution of 1:1000. The cells were then incubated with an Alexa 549-coupled secondary anti-rabbit IgG antibody and the DNA was counterstained with DAPI. Slides were mounted using ProLong Gold (Cell Signaling Technology, Danvers, MA, USA), observed, and recorded using a ZEISS Axio Imager M2 with an X-Cite 120Q light system and Axio Vision software, 2.6 blue edition.

Additionally, an immunofluorescence assay was conducted to quantify the expression of TRPV1 channels in the stimulated OLCs. Briefly, 1 × 10^4^ cells/well were seeded in a 96-well plate containing DMEM supplemented with 10% FBS, 100 U/mL of penicillin, 100 μg/mL of streptomycin, 0.25 μg/mL of amphotericin, and 10 ng/mL of TGF-β1, and adhesion was allowed for 24 h at 37 °C and 5% CO_2_. The cells were stimulated with free CZP, CZP-loaded, or unloaded nanogels diluted in DMEM supplemented with 2% FBS for 1, 3, 5, or 7 days. At different time points, the stimuli were removed and the cells were washed with PBS, fixed with 4% PFA, and stored at 4 °C until processing. The cells were permeabilized with Triton X-100 (Merck Millipore, Burlington, MA, USA) for 10 min at 20 °C and non-specific binding was blocked with 10% goat serum for 30 min at 20 °C. The cells were incubated with the primary anti-TRPV1 antibody (1:1000 diluted) for 1 h at 37 °C, and subsequently with the anti-rabbit secondary antibody coupled to fluorescein isothiocyanate (FITC) for 30 min at 20 °C. The readings were performed using an Infinite 200 PRO Microplate Reader (Tecan, Männedorf, Switzerland) at wavelengths of 495 nm/519 nm. Thereafter, the cells were used for total protein quantification using bicinchoninic acid (BCA; Thermo Fisher Scientific, Waltham, MA, USA) and CuSO_4_; a calibration curve with serial dilutions of bovine serum albumin in 0.15 M NaCl (2 μg/μL–0.03125 μg/μL) was generated for protein quantification. After adding BCA, the samples were incubated at 37 °C for 30 min and absorbance was measured at a wavelength of 570 nm. Normalization was performed using the amount of protein per well and the RFU/μg of protein per well. The experiments were performed in triplicate.

Finally, to determine the functionality of TRPV1 channels, OLCs were seeded on 384 black plates (SPL Life Sciences, Pocheon, Republic of Korea) at a density of 1.5 × 10^3^ cells/well and allowed to adhere for 24 h in DMEM with 10% FBS. Then, the cells were cultured in DMEM with 2% FBS and treated with free CZP, CZP-loaded, or unloaded nanogels for 7 days. At 1, 3, 5, and 7 days, cells were removed, treated with 2 μM of Fluo-4 AM for 45 min, and then washed with PBS after the removal of medium for 30 min to allow for probe de-esterification. The Fluo-4 AM-loaded cells were subsequently stimulated with 100 μM CAP as a TRPV1 agonist, and immediately subjected to fluorescence measurement using a microplate reader (485 nm/520 nm). Cation influx was calculated as the ratio of F/F0, corresponding to the fluorescence of the stimulated loaded cells/fluorescence of the unstimulated loaded cells. The experiments were performed in triplicate.

### 2.10. Statistical Analysis

The results are expressed as means ± standard error and the experiments were performed in triplicate. A Shapiro–Wilk test was conducted to evaluate the data’s normal distribution. For the analysis, a two-way analysis of variance and the Tukey’s test were used to determine statistical significance using GraphPad Prism 8. Statistical significance was set at *p* < 0.05.

## 3. Results

### 3.1. Nanogels Have Desirable Characteristics

**Figure 1:**
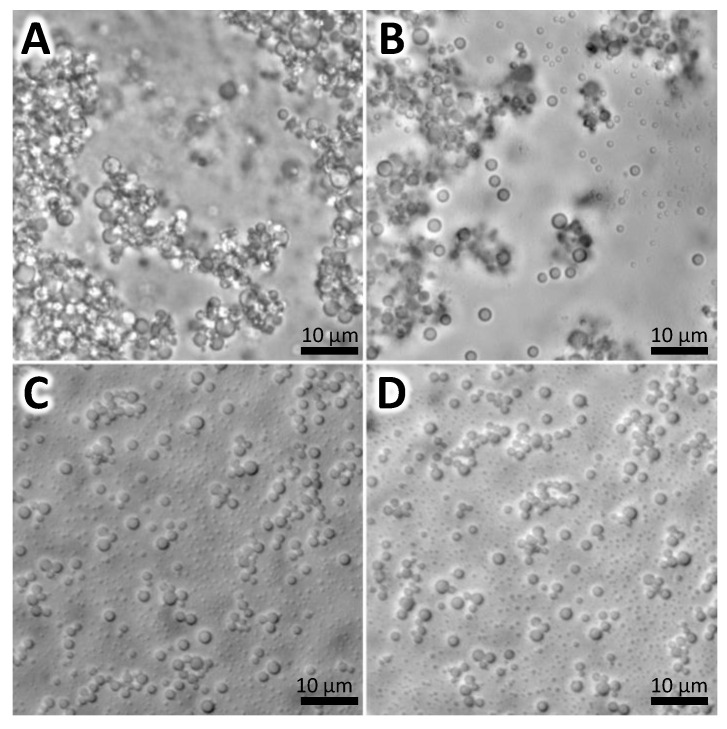
Microscopy images of selected prototype nanogels. (**A**,**B**): Unloaded nanogels. (**C**,**D**): CZP-loaded nanogels. In total, 71.67% of nanogels had a size between 300 and 950 nm.

**Figure 2:**
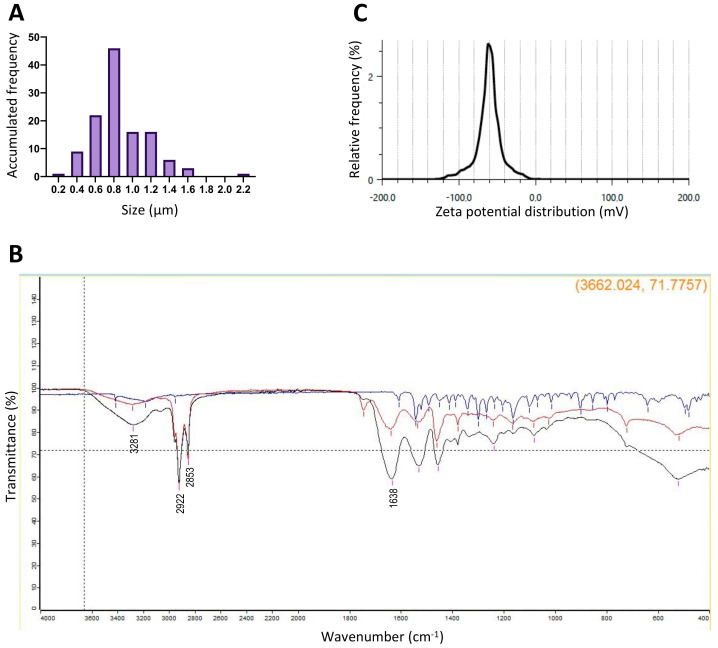
Nanogels characterization. (**A**). Size accumulated frequency of selected prototype; (**B**). FTIR showing the interaction between the nanogels and the active. The line blue corresponds to CZP powder, and the black and red to CZP-loaded and unloaded nanogels, respectively. The absence of new bonds between the active compound and nanogels was maintained; (**C**). Zeta potential distribution [mV] of CZP-loaded nanogels. The unloaded prototypes presented a charge of −59.6 ± 2.06 mV, and in the CZP-loaded prototypes this was −36 ± 3.25 mV.

The emulsification-gelation technique was employed to generate the nanogels (Figure 1). Five prototypes were created, and their respective variations in sizes, PDIs, and yields are summarized in Table 3. The adjustment of certain elaboration parameters, such as surfactant percentage and homogenization amplitude, facilitated a reduction in the particle size of the nanogels (Figure S1). Among the prototypes, variant number 5 was chosen as it exhibited size distribution, with 71.7% of the nanogels ranging between 300 and 950 nm, accompanied by a low PDI of 0.11 ± 0.08 (Figure 2A). The quantification of the active ingredient in the selected prototype was determined using HPLC, revealing a loading efficiency of 67.5 ± 0.04%. Moreover, in vitro enzymatic degradation studies demonstrated a loading efficiency of 63.3 ± 0.17%. A microbiological analysis confirmed the absence of bacterial growth, underlining the safety of nanogels.

For the chosen prototype, FTIR spectroscopy confirmed the chemical stability of the active ingredient (CZP) in the nanogels, as evidenced by the absence of new binding between the active ingredient and the nanogels (Figure 2B). The FTIR spectra of both the CZP-loaded and unloaded nanogels showed characteristic bands corresponding to the distinct functional groups of glutaraldehyde. Notably, the bands at 2922 cm^−1^ and 2853 cm^−1^ corresponded to CH stretching vibrations, while that observed at 3281 cm^−1^ was indicative of amine and amide (N–H) bonding of gelatin (Figure 2B). The crosslinking between gelatin and glutaraldehyde resulted in the formation of a Schiff base located at 1638 cm^−1^.

Regarding the zeta potential, the unloaded prototypes presented a charge of −59.6 ± 2.06 mV, whereas the CZP-loaded prototypes showed a zeta potential of −36 ± 3.25 mV (Figure 2C). These results indicate that the chemical stability of CZP in the nanogels and the absence of new bonds between the active compound and nanogels were maintained, ensuring the effective functionality of the nanogels as drug delivery systems.

### 3.2. Loaded Nanogels Showed Sustained Release of CZP

**Figure 3:**
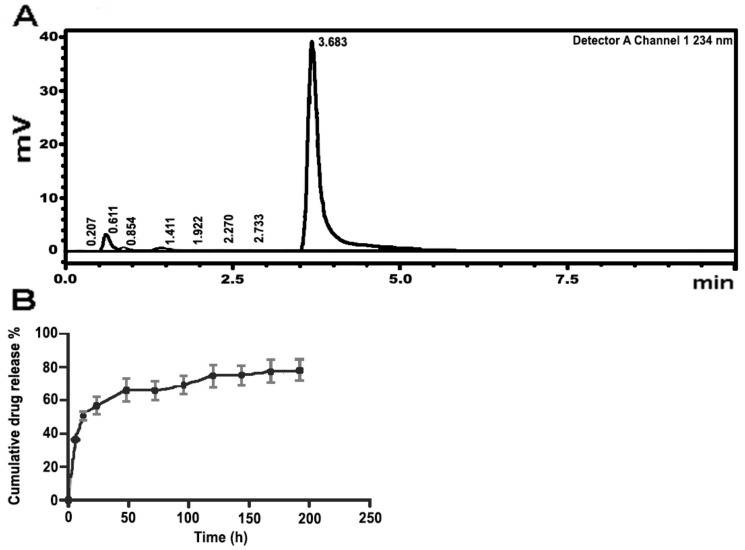
CZP quantification by HPLC. (**A**). HPLC chromatogram of CZP at 234 nm. The retention time was 3.7 min; (**B**). Cumulative CZP release profile for 192 h, the sustained release over the evaluation period is observed. All data are representative of the median of three independent experiments.

CZP release was quantified using HPLC with a calibration curve (R^2^ = 0.9876; Figure S2). The retention time was 3.7 min (Figure 3A). The release profile of CZP from the selected prototype showed a rapid release, with 36.2% being released within the first 6 h, reaching 50.7% at 12 h and maintaining CZP release at later time points throughout the entire evaluation period (Figure 3B). Specifically, the CZP release reached 56.9% at 24 h and 75.1% at 144 h, with a total release of 78.25% by the end of the 192 h assay. These results indicate a sustained release of CZP over time.

### 3.3. CZP-Loaded and Unloaded Nanogels Induce Low Cytotoxicity

**Figure 4:**
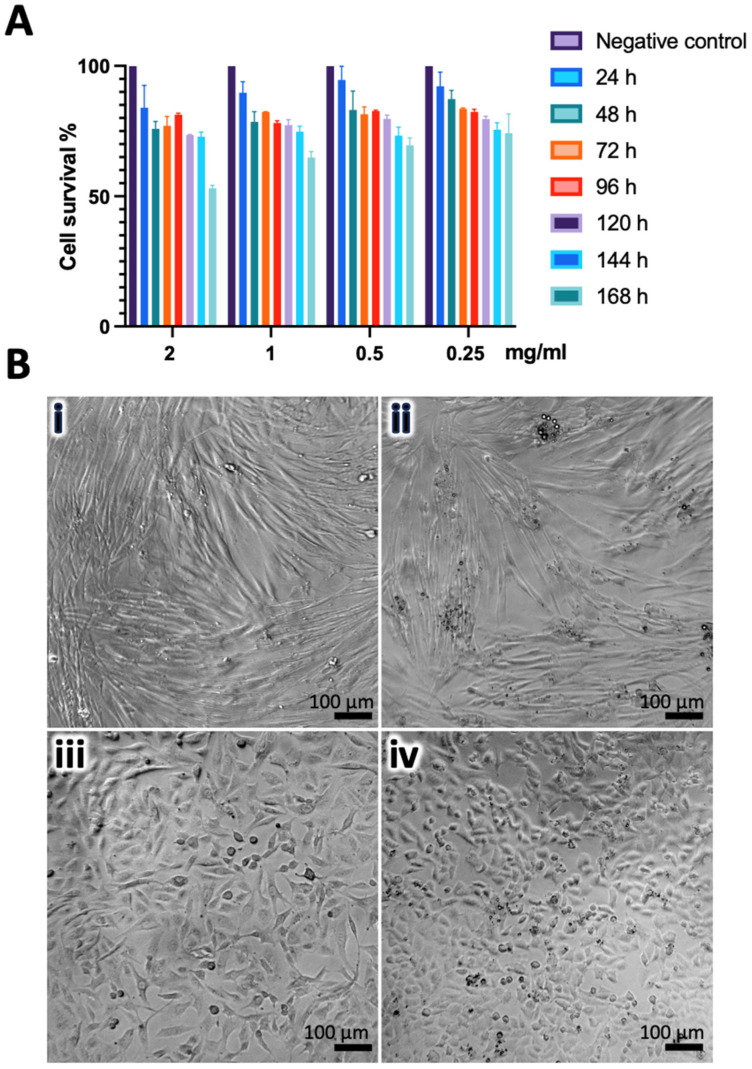
Cell viability after nanogels stimulation. (**A**). Cell survival in OLCs treated with CZP-loaded nanogels for 168 h. (**B**). Phase-contrast microscopy figures, cells treated with 2 mg/mL nanogels, selected prototype. (**i**,**ii**): HGFs, cells treated with CZP-loaded nanogels, and control cells, respectively. (**iii**,**iv**): OLCs, cells treated with CZP-loaded nanogels, and control cells, respectively. Non-substantial structure or shape changes were observed until 168 h post-treatment. All data are representative of the median of three independent experiments.

The viability of the OLCs and HGFs treated with CZP-loaded and unloaded nanogels was assessed over seven days, revealing a consistent viability over 60% at various concentrations (Figure 4A). For example, at 48 h, the OLCs and HGFs treated with 2 mg/mL of unloaded nanogels exhibited viabilities of 81.6 ± 0.36% and 94.5 ± 2.55%, respectively, while cells treated with CZP-loaded nanogels showed values of 75.94 ± 2.85% and 82.4 ± 2.22%.

A microscopic analysis of the cells treated with nanogels at 7 days post-treatment did not reveal notable morphological alterations (Figure 4B). However, observations of the OLCs treated indicated a slight transition towards a more rounded morphology, distinct from the typical fibroblastic-like shape observed in non-treated cells. This difference was not evident in HGFs, suggesting a heightened susceptibility of OLCs to nanogel treatment.

### 3.4. CZP Released from Nanogels Modulated the Expression and Reduced TRPV1 Activation

**Figure 5:**
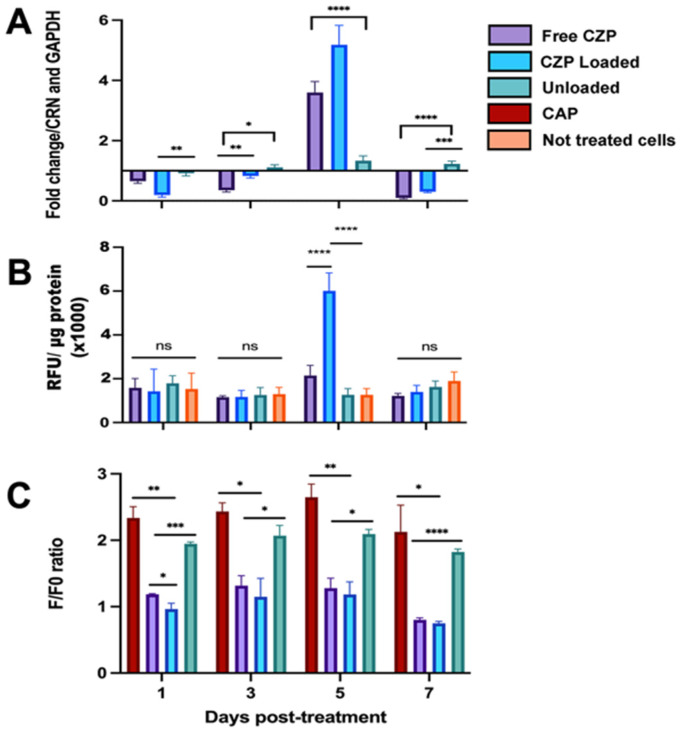
Expression and TRPV1 activity in OLCs treated with free CZP, CZP-loaded, and unloaded nanogels. (**A**). Relative quantification of *TRPV1* transcripts, using the Schefe method and *CRN* and *GAPDH* as reference genes; (**B**). Immunofluorimetric assay for TRPV1. The normalization was performed using the total amount of protein/well and the relative fluorescence units (RFU)/μg of protein/well. (**C**). Calcium fluxes using Fluo-4 AM probe. The cells were treated with 100 μM of CAP, 40 μM of CZP in solution + CAP, CZP loaded + CAP, or unloaded nanogels + CAP and the measurement of fluorescence intensity by microplate reader was performed. The data points are representative of the mean of three independent experiments. All bar plots and error bars denote mean ± SD. *P*-values were calculated using a two-way ANOVA and Tukey test (ns: *p* > 0.05, * *p* ≤ 0.05; ** *p* ≤ 0.01; *** *p* ≤ 0.001; and **** *p* ≤ 0.0001).

**Figure 6:**
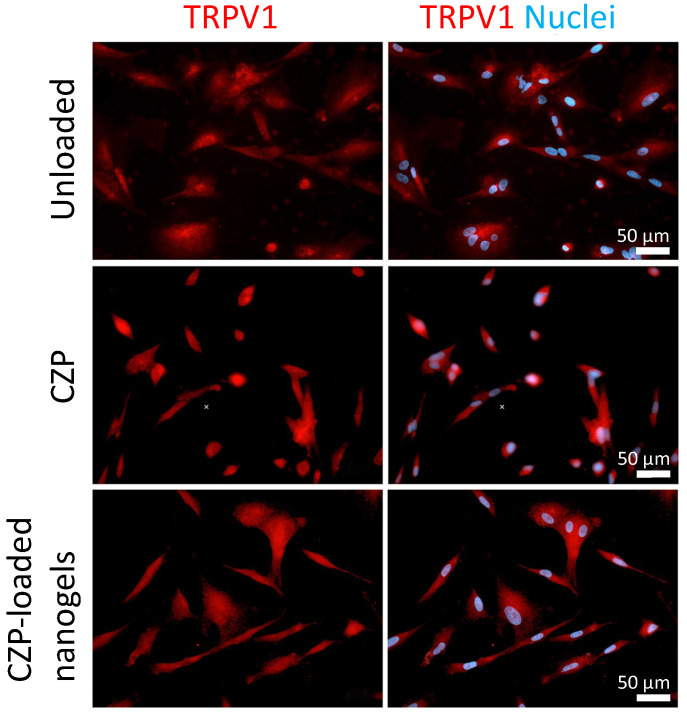
Expression of TRPV1 by immunofluorescence in OLCs stimulated with the nanogels. OLC treated with unloaded nanogels, CZP in solution, or CZP-loaded nanogels. Specific antigen and nuclear markers are shown in red and blue, respectively. No differences were observed among the various treatments.

The expression of *TRPV1* transcripts in the stimulated OLCs was downregulated at days 1, 3, and 7 in the cells treated with CZP-loaded nanogels and CZP in solution compared to the controls (Figure 5A). However, on the fifth day post-treatment, there was a significant increase in *TRPV1* transcripts in the cells treated with CZP in solution or CZP-loaded nanogels (*p* < 0.0001). Although the channel protein was similarly detected by fluorescence in the cells stimulated with the different treatments (Figure 6), the immunofluorimetric assay on the fifth day showed a significant increase in the cells treated with the CZP-loaded nanogels (6012 RFU/μg protein) compared to the negative controls (1261 RFU/g protein) (*p* < 0.0001), with no differences at other post-treatment days (Figure 5B).

TRPV1 activation in the OLCs, evaluated by calcium flux (Figure 5C), was reduced in the cells treated with CZP in solution + CAP or CZP-loaded nanogels + CAP. For example, on the fifth day of treatment, the calcium influx showed a decrease from 2.65 ± 0.20 in the stimulated CAP cells to 1.28 ± 0.15 and 1.18 ± 0.18 in those co-stimulated with CZP in solution + CAP (*p* = 0.0093) or CZP-loaded nanogel + CAP, respectively (*p* = 0.0088). A comparison of the CZP-loaded nanogel + CAP and free CZP + CAP groups revealed a statistically significant reduction in calcium influx on day 1 post-treatment (*p* = 0.048). However, no significant differences were found thereafter.

Regarding the cells stimulated with unloaded nanogel + CAP, a higher F/F0 ratio was observed (1.95 ± 0.02, 2.07 ± 0.16, 2.09 ± 0.06, and 1.82 ± 0.04 at 1, 3, 5, and 7 days, respectively), compared with that of free CZP + CAP (1.19 ± 0.009, 1.32 ± 0.15, 1.28 ± 0.15, and 0.80 ± 0.03), or CZP-loaded nanogel + CAP (0.95 ± 0.08, 1.15 ± 0.28, 1.18 ± 0.19, and 0.75 ± 0.02) on all evaluation days. No differences were observed in the intracellular calcium levels between the cells stimulated with CAP and those treated with unloaded nanogel + CAP. Thus, this study demonstrated the formulation of biocompatible and biodegradable CZP-loaded nanogels, which effectively modulate the activity of TRPV1 on OLCs by releasing a synthetic antagonist over an extended period.

## 4. Discussion

The management of dental pain is a common challenge in dental practice. The therapeutic inhibition of TRPV1 activity through targeted antagonists has exhibited promise in managing both acute and chronic dental pain [2], for instance, through the local application of formulations capable of obliterating dentinal tubules. While TRPV1 antagonists have been associated with adverse effects, others are currently in clinical trials [5,31]. There is growing interest in developing biomaterials loaded with drugs for the local treatment and prevention of dental diseases. These materials have significantly improved the effectiveness of treatment, reduced the associated side effects related to the systemic use of drugs, and addressed issues such as constant salivary flow [32]. The development of micro/nanoencapsulants with unique structures and properties has facilitated the creation of more effective drug release platforms. This offers the potential for the localized management of various dental pathologies and infections, as well as addressing symptoms such as pain and oral manifestations of systemic diseases [33,34].

The potential use of nanocarriers for loading various active molecules to aid in the diagnosis or treatment of multiple pathologies is widely acknowledged [13]. This study presents the fabrication of gelatin nanogels within an established size range (10–999 nm) [35] using an emulsion-gelation technique. This method offers a simple, easy, and reproducible method suitable for laboratory use and potential scalability [21,36]. Importantly, modifying the surfactant percentage and homogenization conditions resulted in a reduction in particle size in various nanogels prototypes elaborated in this study. These adjustments enabled the selection of a prototype with the appropriate particle size through particle stabilization. Such parameters affecting particle size have been described elsewhere [37]. The selected prototype exhibited an appropriate size and biocompatibility, suggesting its potential for releasing active agents into the oral cavity. The calculated PDI (0.11 ± 0.08) indicates that the nanogels have a moderately homogeneous size distribution and monodisperse colloidal particles (values less than 0.1), which are essential factors for a good performance and their potential applications as drug delivery system [26].

The natural polymer used to fabricate the nanogels, gelatin B, is obtained through the basic hydrolysis of collagen. Gelatin B, with a pH range of 4.7–5.6, acquires negative charges during preparation at physiological pH 7.4, which explains the values obtained by the zeta potential assay. However, the increase in zeta potential of the loaded nanogels could be related to the basic nature of CZP (pKa 9.2) [38]. Other formulations using gelatin, such as buccal patches, have been proposed for the administration of local anesthetics in buccal treatments, highlighting the broad potential use of gelatin in this field [39].

The drug release pattern observed with this formulation aligns with that reported for gelatin nanoparticles, where a biphasic model was described. Such release pattern is characterized by a significant burst effect owing to the immediate release of the free drug associated with the surface, followed by a sustained release in the later stages by slow diffusion [40]. Several mechanisms have been proposed for the release of the active molecule from the nanogel, including release by erosion or degradation, self-diffusion through the pores, and the cleavage of the gelatin matrix by proteolytic enzymes [16,41], which could explain the release of CZP up to 192 h of evaluation. The release of a significant fraction of the active drug within the initial stage is known as the burst effect, estimated to correspond to approximately 30% of the load for nanocarrier systems [42]. In this study, the CZP release obtained within the first 6 h corresponded to 36.2% of the load.

The cytotoxicity analysis revealed a viability range of 60–90% in OLCs treated with both the selected prototypes of CZP-loaded and unloaded nanogels during the 168 h evaluation period. In HGFs, the survival rate also remained above 60% throughout the evaluation. No substantial changes were noted in the monolayer cells by light microscopy, nevertheless, the treated OLCs showed a slight rounded appearance. Similar findings were reported by Pham et al. [43], who observed the typical morphology of healthy human dermal fibroblasts when stimulated with a hybrid thermosensitive hydrogel containing gelatin for up to 72 h after treatment. Another report indicates that OLCs treated with dental resin monomers promptly respond to maintain cell homeostasis, exhibiting alterations in cell membrane integrity, metabolic activity, and survival, such as an irregular morphology and partial detachment from the monolayer [44]. To validate the metabolic changes, membrane damage, activation of the apoptotic pathway, mitochondrial activity, and other effects induced by nanogels in OLCs, further assays must be developed.

The duration of the pharmacological treatment depends on various patient factors, especially concerning the potential application of nanogels for managing acute dental pain. Some studies have suggested that the short-term use of analgesic medications (first 72 h) is sufficient for managing postsurgical pain [45], while others conditions require longer periods for procedures with more severe pain [46]. The release profile obtained in this study showed the controlled release of the loaded molecule up to day 8 of evaluation, suggesting that it could be appropriate to maintain the amount of active ingredient in the target. Further in vivo and clinical trials are required to confirm this hypothesis.

Although no significant differences were observed between the biological responses generated by the free CZP and CZP-loaded nanogels, the lower activity induced by the free CZP group may be attributed to the longer contact time of CZP with cells and the culture medium. This prolonged exposure could induce some metabolic processes, decreasing the antagonistic effect on the receptor in comparison with the CZP released from nanogels, which are delivered over an extended period. Studies have shown that the use of actively loaded nanocarriers generates greater cellular activity. For example, Jimenez-Lopez et al. [47] found that the antitumor effect of paclitaxel (PTX) nanoparticles was far superior to that of free PTX against lung cancer cells in culture. Similarly, Ulmansky et al. [48] demonstrated that nanoliposomes with glucocorticoids (GC) suppressed arthritis significantly compared to higher doses of parenterally administered free GC.

A reduction in the expression of TRPV1 in cells treated with CZP was observed on day 7 compared to day 5. Some authors have reported the downregulation of different receptors after chronic stimulation with an antagonist. For example, the internalization of 5-HT2A receptors following antagonist exposure has been demonstrated in in vivo and in vitro models [49]. The internalization of TRPV1 could be a possible explanation for this phenomenon, and the lower influx of calcium on day 7 may have been related to a decrease in TRPV1 expression.

A limitation of this study is it being technical in nature. We attempted to obtain ultrastructural information on the shape and size of our nanogels using SEM. Yet, the high water content of the nanogels, typically ranging from 70% to 99%, resulted in a challenge in maintaining their original form during the SEM processing, which required drying the samples. This technical challenge has been previously reported [50].

Nanodentistry, a pioneering field in oral health care, explores the use of nanomaterials for the treatment, diagnosis, pain relief, and prevention of oral and dental diseases, gaining attention in recent years [51,52]. The CZP-loaded nanogels discussed in this study offer insights into the potential development of new formulations loaded with diverse drugs that target sites with difficult access in the mouth, providing a greater drug efficacy and fewer side effects for managing various pathologies, including dental pain, periodontal and mucosal diseases, and oral cancer, among others. However, in vivo studies are necessary to evaluate the possible role of these nanogels in the management of oral conditions.

## 5. Conclusions

In this study, a novel nanogel formulation was developed using biodegradable and biocompatible polymers. Our results demonstrate that the controlled release of a TRPV1 antagonist over an extended period effectively modulates the expression and activation of the channel. The nanogels exhibit a low in vitro cytotoxicity. The versatility of this formulation allows for the loading of various active ingredients, enabling controlled release and making it a promising model for dental research.

Further research is required to fully understand the mechanisms of action of these nanogels and optimize their therapeutic efficacy. This includes conducting in vivo studies to evaluate the potential of these nanogels in managing various dental conditions and investigating the impact of different formulations and release profiles on patient outcomes.

## Figures and Tables

**Table 1:** Conditions for nanogels elaboration.

Prototype	Disperse Phase *	Continuous Phase (Surfactant)	Homogenization Method		
			Magnetic Agitation	Ultrasonic Homogenization (Amplitude, Time)	High-Shear Homogenization
1	0.25 g gelatin topped with H_2_O to 5 g	25 g mineral oil (Sorbitan 1%)	1000 rpm	50%, 3 min	NA
2	0.25 g gelatin, topped with H_2_O to 5 g	25 g mineral oil (Sorbitan 1%)	1000 rpm	50%, 6 min	NA
3	0.25 g gelatin, topped with H_2_O to 7.5 g	25 g mineral oil (Sorbitan 2.5%)	1000 rpm	70%, 10 min	NA
4	0.25 g gelatin, topped with H_2_O to 7.5 g	25 g mineral oil (Sorbitan 7.5%)	1000 rpm	70%, 10 min	13,800 rpm, 15 min
5	0.25 g gelatin, topped with H_2_O to 7.5 g	25 g mineral oil (Sorbitan 10%)	1000 rpm	70%, 10 min	13,800 rpm, 15 min

* Crosslinker: Glutaraldehyde 0.1% *w*/*w*. NA: not applicable.

**Table 2:** Oligonucleotides used for RT-qPCR assay.

Gene	Primer Sequences (5′-3′)	Fragment Size
*TRPV1*	Forward-GTGCACTCCTCGCTGTACGA Reverse-CACCTCCAGCACCGAGTTCT	(66 bp)
*GAPDH* *CRN*	Forward-CACTAGGCGCTCACTGTTCTC Reverse-AAATCCGTTGACTCCGACCT Forward-CAATGCTGACGGCATGTACGA Reverse-CACGAACGGAACTTCATGGTG	(90 bp) (165 bp)

**Table 3:** Characteristics of different prototypes.

Prototype Number	Average Size (µm)	PDI	Yield (%)
1	8.41 ± 1.65	0.31	28.11 ± 1.40
2	4.22 ± 1.44	0.12	43.37 ± 4.34
3	2.97 ± 1.15	0.15	57.91 ± 3.25
4	1.07 ± 0.45	0.18	59.60 ± 4.47
5	0.85 ± 0.11	0.11	69.23 ± 8.37

## Data Availability

Data are contained within the article.

## Supplementary Materials

The following supporting information can be downloaded at: https://www.mdpi.com/article/10.3390/pharmaceutics16030355/s1, Figure S1. Microscopy images of several nanogel prototypes, Figure S2. Calibration curve used to quantify CZP by HPLC.
