# Supplementary material for: Modulation of TRPV1 on Odontoblast-like Cells Using Capsazepine-Loaded Nanogels
Source: Pharmaceutics. 2024 Mar 3;16(3):355. doi: 10.3390/pharmaceutics16030355 (PMC10975074; doi:10.3390/pharmaceutics16030355)
Supplement: Supplementary file 1 [file pharmaceutics-16-00355-s001.zip › pharmaceutics-2819415-supplementary.pdf]

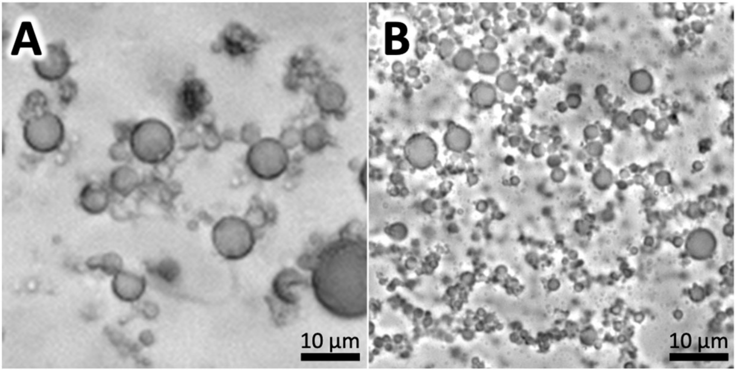

Figure S1. Microscopy images of several nanogel prototypes are shown: A. Prototype number 3, and B. Prototype number 4. Adjusting the surfactant percentage and homogenization amplitude facilitates the reduction in the particle size of the nanogels.

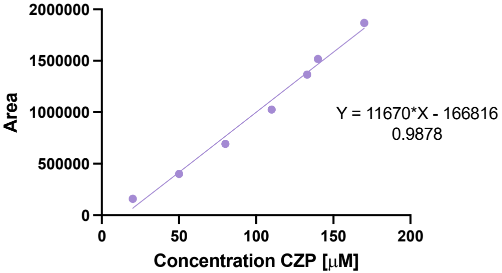

Figure S2. Calibration curve used to quantify CZP by HPLC. Serial dilutions of CZP were done and analyzed by HPLC (20-50-80-110-140 and 170 µM). The peak area and CZP concentration were used to develop the calibration curve. Image elaborated using GraphPad Prism 8.
